# Environmentally-Friendly Dense and Porous Geopolymers Using Fly Ash and Rice Husk Ash as Raw Materials

**DOI:** 10.3390/ma9060466

**Published:** 2016-06-14

**Authors:** Daniele Ziegler, Alessandra Formia, Jean-Marc Tulliani, Paola Palmero

**Affiliations:** Department of Applied Science and Technology, INSTM Research Unit Polito, Lince Laboratory, Politecnico di Torino, Corso Duca degli Abruzzi 24, Torino 10129, Italy; daniele.ziegler@polito.it (D.Z.); alessandra.formia@polito.it (A.F.); jeanmarc.tulliani@polito.it (J.-M.T.)

**Keywords:** geopolymers, mechanical properties, microstructure, porosity

## Abstract

This paper assesses the feasibility of two industrial wastes, fly ash (FA) and rice husk ash (RHA), as raw materials for the production of geopolymeric pastes. Three typologies of samples were thus produced: (i) halloysite activated with potassium hydroxide and nanosilica, used as the reference sample (HL-S); (ii) halloysite activated with rice husk ash dissolved into KOH solution (HL-R); (iii) FA activated with the alkaline solution realized with the rice husk ash (FA-R). Dense and porous samples were produced and characterized in terms of mechanical properties and environmental impact. The flexural and compressive strength of HL-R reached about 9 and 43 MPa, respectively. On the contrary, the compressive strength of FA-R is significantly lower than the HL-R one, in spite of a comparable flexural strength being reached. However, when porous samples are concerned, FA-R shows comparable or even higher strength than HL-R. Thus, the current results show that RHA is a valuable alternative to silica nanopowder to prepare the activator solution, to be used either with calcined clay and fly ash feedstock materials. Finally, a preliminary evaluation of the global warming potential (GWP) was performed for the three investigated formulations. With the mix containing FA and RHA-based silica solution, a reduction of about 90% of GWP was achieved with respect to the values obtained for the reference formulation.

## 1. Introduction

The European Community has recently issued a series of regulations to limit the energy consumption and the exploitation of natural resources, besides promoting the use of secondary raw materials and minimizing the amount of wastes disposed in landfills.

Working in this direction, the present study is focused on the development of dense and porous alkali-activated materials for the building sector, mainly realized by waste products.

In fact, the EU Community purpose of cutting greenhouse emission by 80%–95% by 2050 [1] has a serious implication for the construction industry, being one of the greatest consumers of energy, resources and raw materials. In Europe, buildings through their life cycle, including construction, operation and demolition consume approximately 50% of the total energy demand and contribute almost 50% to the CO_2_ emissions released in the atmosphere [2]. In this frame, the EU has recently put forward a package to support the transition to a circular economy [3], in which the value of products and materials is maintained for the longest time as possible. In this economic scheme, wastes and resources are minimized, whereas the use of secondary raw materials is encouraged to create further value. To this aim, the aspect of “sustainable use of natural resources” has been recently introduced in the European Construction Product Regulation (ECPR), which is laying down harmonized conditions for the marketing of construction products. To fulfil this request, the use of environmentally-compatible raw and secondary materials is becoming a fundamental need [4].

In the last few years, the research has focused on the development of green alternatives to Ordinary Portland Cement (OPC), in terms of the limitation of the CO_2_ footprint and conscious use of natural resources. On the ground of the good results achieved, growing attention has been paid to the family of alkali-activated binders and in particular to the geopolymers. These materials have shown to offer an environmentally-friendly, technically-competitive alternative for both mechanical [5] and durability properties [6,7,8] to OPC and are beginning to be used in several applications [9,10,11].

The geopolymerization process starts with the dissolution of Al and Si (from the Al-Si-rich materials) in alkali solutions as hydrated reaction products with NaOH or KOH, hence forming a [M_x_(AlO_2_)_y_(SiO_2_)_z_∙nMOH∙mH_2_O] gel (M: Na or K) [12]. Several silico-aluminates powders, such as kaolinitic clays [13], metakaolin, fly ash [14,15], blast furnace slag and mixtures of them, could be used as precursors of geopolymerization [12,16]. The most important parameters that affect the mechanical properties of the final product are the reactive silica content, the Si/Al ratio, the amorphous phase content and the calcium content [17,18,19].

Metakaolin-based geopolymers can be manufactured consistently with predictable properties due to the common features of the powder, which is commercially available worldwide [16]. Curing is normally carried out at low temperature (20–120 °C) for 3–10 h, depending on the raw material and the composition used. The complete development of mechanical properties generally takes place after five days and is strongly related to the Si/Al ratio, as previously mentioned [12,17,18,19,20,21,22]. The lack of production standards resulted in a wide range of data related to the mechanical properties, since the compressive strength ranges from 6–60 MPa [16].

As an alternative to metakaolin, fly ash (FA) can be used as well. FA is an industrial waste with pozzolanic properties, obtained from thermal power plants. Differently from the metakaolin, FA does not derive from a well-defined starting material. Even if the predominant network of the final material is composed of silicon and aluminum, the presence of impurities (CaO, iron, blast furnace slag) plays an important role in modifying setting time, slump, strength and shrinkage [16]. Due to the high levels of amorphous silica and alumina, FA are often used to produce geopolymers with interesting properties [16,17]. In addition, the curing conditions play an essential role in the development of FA-based geopolymers properties, as shown in the work of Kovalchuk *et al.* [23], in which an excellent mechanical strength (*R*_ck_ up to 102 MPa) was reached for samples cured in covered molds at 95 °C for 2 h.

Concerning the environmental impact of geopolymers, the available literature data are highly dispersed [9,16,24,25] depending on the raw materials and alkali activator used. FA is particularly interesting from the environmental point of view, since it is an industrial waste and does not require any energy input apart from transportation; on the opposite side, when clay is used as the raw material, it requires a calcination process at high temperature (≈650–750 °C) to dehydrate the powder and improve its reactivity in the geopolymerization process.

Concerning the alkaline activator, sodium silicate solution, which is frequently modified by adding extra amorphous silica nanopowder [26] and sodium or potassium hydroxide, is commonly used to prepare geopolymers. However, sodium silicate is manufactured through an energy-intensive process, involving the calcination of sodium carbonate and quartz sand at temperatures ranging between 1400 and 1500 °C [27]. Therefore, in order to reduce the environmental impact of the geopolymers, it is necessary to properly select the feedstock material and to limit the amount of sodium silicate in the mixture or to substitute it by less environmentally-impacting solutions, but still containing reactive amorphous silica. In this context, a point of interest is silica-rich wastes, such as rice husk ash (RHA). RHA is an industrial waste, generated by the combustion of rice husk (biomass used in co-generation plants for the production of green energy [28]) and contains around 85%–90% SiO_2_ mainly in the amorphous form [12]. This waste has been used for the production of refractory materials, to synthesize silicon carbide, as a supplementary cementitious material for its pozzolanic features [29,30,31,32,33,34], as a component in the production of alkali-activated binders [35] and, finally, as a point of interest in this contribution, as a precursor for the production of soluble sodium silicate [36,37]. Starting from our previous interesting results achieved with metakaolin-based geopolymers, activated with sodium and potassium silicate solutions [38], this study assesses the feasibility to use two industrial wastes as raw materials for the production of geopolymer pastes, namely RHA and FA, on the ground of previous literature [39]. As RHA is a suitable source of amorphous silica, this research aims to eliminate the use of commercial sodium/potassium silicate solution and of silica nanopowder that are generally used for geopolymer synthesis. Thus, RHA was dissolved in a KOH solution and used as an economical and green alternative to the commercial potassium silicate solution. Similarly, metakaolin was substituted with FA powder, which is a Si-Al-rich waste product and does not require any high temperature treatment prior to using.

Therefore, in this study, three typologies of dense samples were produced: (i) a calcined clay activated with potassium hydroxide and nanosilica, used as reference samples; (ii) calcined clay activated with RHA dissolved into KOH solution; and (iii) FA activated with the alkaline solution realized with RHA. The last formulation, containing FA as feedstock and RHA as the silica source for the silicate solution, can be considered particularly interesting because of the high recycled content. Furthermore, starting from the last two compositions, porous samples were realized, by surveying the role of aluminum powder on the foamability of the samples. The development of such porous materials was motivated by the increasing interest in lightened geopolymers, due to their high potential in the development of thermal insulator elements. For instance, Zhao *et al.* [40] developed fly ash-based geopolymer foams, showing a significantly low thermal conductivity (0.084 W/mK) and an excellent resistance when exposed to fire temperatures up to 1150 °C for 2 h. The development of new, thermal insulating building elements has great interest, since their use constitutes the most effective way of reducing heat losses in buildings, thus reducing the energy needs. These elements represent a very profitable business sector for the building material industry, which is destined for a further expansion in view of the achievement of the target of zero energy buildings [1].

In our work, the porous samples realized with FA as the feedstock and RHA as the silica source for the silicate solution appear to be very innovative materials, without any previous reference in the literature and could be considered particularly interesting because of the high recycled content and of their physical-mechanical features. Finally, to quantify the benefit on the environmental impact associated with the use of waste materials in the studied samples, a preliminary evaluation of the global warming potential was done for the three formulations.

## 2. Materials and Methods

Geopolymers were realized using halloysite clay and fly ash as starting materials.

Halloysite clay, supplied by Applied Minerals Inc., was calcined at 650 °C for 3 h to dehydrate the hydroxide groups and to produce an almost amorphous powder (referred to as HL).

Fly ash (FA) was obtained from the ICIC/Enel SpA coal-fired power plant of La Spezia, Italy. It is characterized by an absolute density of 2.3 g/cm^3^ and a water solubility of 1 g/L. Rice husk ash (RHA), a by-product of a biomass combustion plant (Idroblins srl, Crova, Italy, [28]), was also investigated as a raw material for preparing the alkaline solution. It is a dry black powder, with a moisture content lower than 1% and specific weight of 0.17–0.24 g/cm^3^.

X-ray diffraction (XRD) analysis was used to characterize the as-received powders and geopolymers. Spectra were recorded on a Philips PW 3800 apparatus operating with Cu Kα radiation (0.1541874 nm) and were acquired in the range 5°–70° 2θ, with a step size of 0.05° 2θ and an acquisition time per step of 5 s. Diffraction patterns were indexed by using the Powder Data File database (P.D.F. 2000, International Centre of Diffraction Data, Newtown Square, PA, USA).

X-ray fluorescence (XRF, Rigaku ZSX 100E, Tokyo, Japan) analysis was carried out on the powders, in order to assess their chemical composition.

The inductively-coupled plasma-atomic emission spectrometer (ICP-AES, Perkin Elmer, Optima 7000 DV, Waltham, MA, USA) was used to investigate the dissolution degree of RHA. First, the chemical composition of the starting RHA, sieved under 125 mesh, was determined. The powder was submitted to acid digestion in a microwave oven. Sample aliquots of 100 mg were treated with a mixture of 5 mL of aqua regia and 2 mL of hydrofluoric acid in polytetrafluoroethylene (PTFE) bombs. Four heating steps of 5 min each (250, 400, 600, 250 W power, respectively) followed by a ventilation step of 25 min, were applied. Then, 700 mg of boric acid were added, and the bombs were further heated for 5 min at 250 W, succeeded by 15 min of ventilation. At the end of the full treatment, the samples appeared completely dissolved.

The article size distribution of raw materials was determined by means of a laser granulometer (Fritsch Analyzette 22, Idar-Oberstein, Germany) after dispersion in ethanol and sonication in an ultrasonic bath for 10 min.

Three typologies of dense pastes were prepared, as reported in Table 1, which also gives the designations used. In this study, HL-S is used as the reference material to assess the properties of the other two more innovative, waste-containing compositions, *i.e.*, HL-R and FA-R.

In order to prepare the alkaline silicate solutions, potassium hydroxide (KOH in pellets, analytical grade) and SiO_2_ nanopowder (10–20 nm, 99.5% purity), all supplied by Sigma-Aldrich, were employed. The potassium silicate solution was prepared by adding amorphous silica nanopowder to a clear potassium hydroxide solution (8 M), kept under magnetic stirring for 24 h. After this stirring time, a clear solution was obtained, free from precipitates or silica particle residues, suggesting the complete dissolution of the silica nanoparticles. The SiO_2_/K_2_O and H_2_O/K_2_O molar ratios were fixed at 1.66 and 11, respectively, according to a previous work [38] and to literature data [20,41].

A second alkaline solution was prepared using RHA instead of nanosilica powder: the ash was sieved under 125 mesh and added to a potassium hydroxide solution (8 M) in the same amount of silica nanopowder (leading to a ratio RHA:KOH:H_2_O in the solution equal to 1:1:2). In order to assess the best dissolution condition of RHA into the alkaline medium, three different solubility tests were carried out. Precisely, the RHA/KOH solution was stirred for 4, 24 or 168 h at room temperature. After each test, the solution was separated from the (eventual) precipitate and submitted to ICP-AES analysis. In this case, the suspensions were filtered, and the supernatants were diluted (1:100), stabilized with HNO_3_ (0.1%) and submitted to ICP-AES analysis. After that, calibrations with standard solutions prepared in aliquots of sample blanks were performed.

All of the dense geopolymer pastes were prepared by mechanically mixing raw materials (HL or FA) and the alkaline solution (KOH + SiO_2_ or KOH + RHA) for about 15 min at 200 rpm, before casting into plastic prismatic molds (size of 20 × 20 × 80 mm^3^). The mix design of the different compositions (expressed as wt % of each component in the mixture) is summarized in Table 2.

Once the proper ratio between the components in the solutions was fixed (a SiO_2_/K_2_O molar ratio of 1.66 and a H_2_O/K_2_O molar ratio of 11, as previously explained), the ratio between the raw material and the potassium silicate solution was fixed on the ground of the paste workability, in order to have slurries sufficiently flowing to be easily cast, thus not requiring a de-airing process. Specifically, the apparent viscosity of the pastes was determined by means of a viscometer (Brookfield HBDV-II, Middleboro, MA, USA) and measured at 200 rpm (which corresponds to the mixing rate).

Starting from the two formulations containing industrial wastes (HL-R and FA-R), porous samples were also produced. To this aim, aluminum powder, supplied by Alfa Aesar^®^, Karlsruhe, Germany, was used as the foaming agent and added at 0.05%–0.3% with respect to the total mass, as reported in Table 2. The Al powder is characterized by 99.5% of purity and an average particle size of 7–15 μm, as declared by the supplier. After foaming agent addition, the slurry was mixed at high rpm for 1 min and then cast in prismatic molds. The foamed samples followed the same curing schedule as the dense materials.

The HL-based samples were cured in a sealed container at room temperature, whereas the samples realized with FA were cured at 50 °C for 24 h and then stored at room temperature until testing.

Bulk density was measured in accordance with EN 12390-7, after 14 days of curing.

Flexural strength of the geopolymer samples was measured in three-point-bending, using an electromechanical testing system (Zwick Roell 2014, Ulm, Germany) with a maximum load capacity of 1 kN and standard length. Compressive strength was measured on the far edge of both residual pieces obtained from flexural tests according to the EN 196-1 standard, using a 50-kN closed-loop universal press by Zwick Roell, working in displacement control mode. Dense and foamed samples were submitted to mechanical tests after different curing times (7, 14, 21 and 28 days).

Field emission-scanning electron microscope (FE-SEM, Hitachi S4000, Tokyo, Japan) observations were performed on raw materials and on geopolymer pastes (observations were carried out on the fracture surfaces). The samples were gold sputtered (SPI, West Chester, PA, USA) prior to observations.

## 3. Results and Discussion

### 3.1. Raw Materials’ Characterization

The chemical composition of HL, as assessed by XRF analysis, is reported in Table 3 and shows a predominant fraction of silicon and aluminum oxides, whose content is about 54 wt % and 44 wt %, respectively.

The chemical composition of FA, determined by XRF, is reported in Table 4. The ash is characterized by a high SiO_2_ amount (about 60%), followed by a lower, but still important amount of Al_2_O_3_ (25%). Non-negligible amounts of Fe_2_O_3_, CaO and K_2_O were also present.

The RHA chemical composition is reported in Table 5 showing that SiO_2_ was the major component of this by-product.

**Figure 1:**
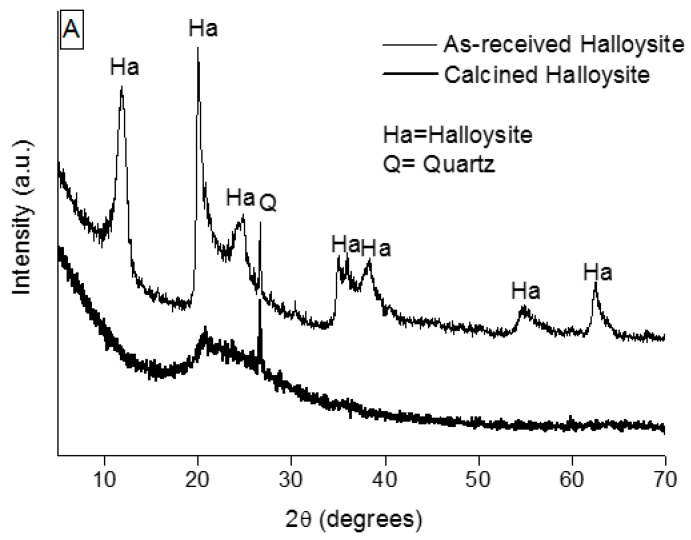

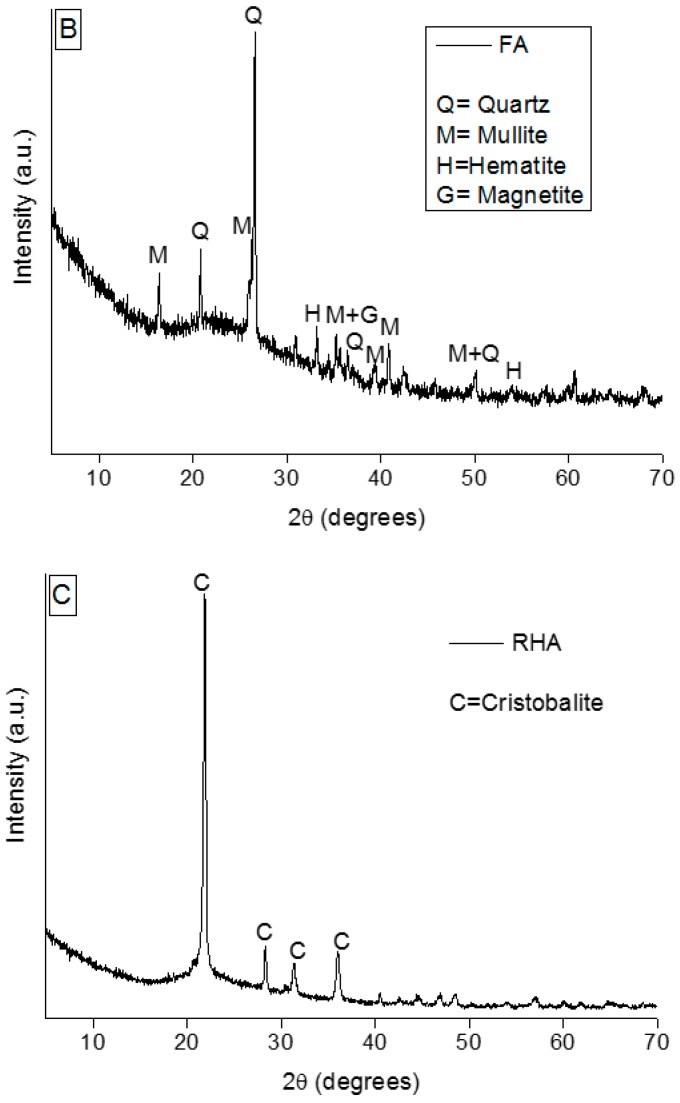
XRD patterns of: (**A**) as-received (thin line) and calcined (thick line) halloysite (HL); (**B**) fly ash (FA); (**C**) rice husk ash (RHA).

The XRD patterns of the starting materials are displayed in Figure 1.

The as-received clay powder contains a significant fraction of crystalline phases: as shown in Figure 1A (thin line), it is possible to identify halloysite (JCPDF No. 09-0451), kaolin (JCPDF No. 75-1593) and quartz (JCPDF No. 83-2468) phases. After calcination at 650 °C for 2 h, the HL powder (thick line) is characterized by a broad band, typical of the amorphous phase, attesting dehydroxylation and thermal decomposition of the previous crystalline phases. Here, only the XRD signals of the quartz phase can be detected, this phase being stable up to about 1000 °C.

In Figure 1B, the XRD pattern of FA is shown: the powder was basically constituted by a vitreous phase (halo registered between 20° and 35° 2θ) and some crystalline phases identified as quartz (JCPDF No. 83-2468), mullite (JCPDF No. 79-1454), hematite (JCPDF No. 79-1741) and magnetite (JCPDF No. 79-0471).

The XRD pattern of the RHA is shown in Figure 1C: it is characterized by a weak band centered around 22° 2θ accompanied with strong cristobalite peaks (JCPDF No. 82-1403). The partial crystallization of the RHA could be related to a high combustion temperature. It was found, in fact, that the silica contained in the ash was partially crystalline when the firing temperature was higher than 800–900 °C, whereas when the pyrolysis was carried out in the range 450–700 °C, the silica was predominantly amorphous [29,42].

**Figure 2:**
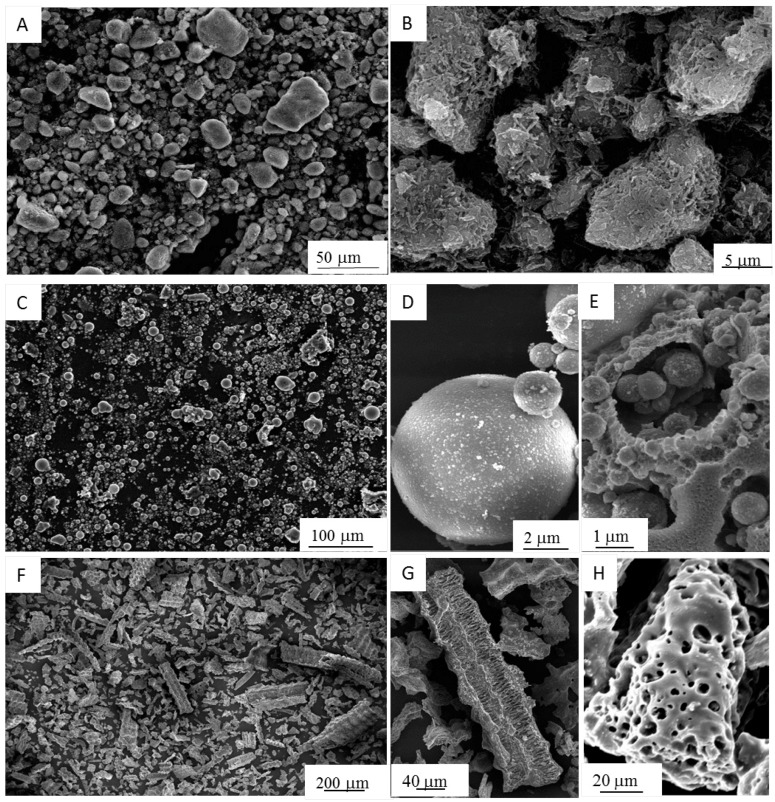
FESEM micrographs of raw materials: (**A**,**B**) calcined HL; (**C**–**E**) FA; (**F**–**H**) RHA.

In Figure 2, the FESEM micrographs of the three powders are depicted. In Figure 2A,B, the morphology of HL is shown. The presence of agglomerates having an almost rounded morphology, whose size ranges between a few and 40 μm, is clearly evident. The higher magnification image (Figure 2B) highlights the hollow tubular morphology of the halloysite primary particles [43], with a length ranging from about 200 nm to 2 μm and diameters close to 100 nm.

The features of the as-received FA particles are shown in Figure 2C–E. By the lower magnification image (Figure 2C), two different morphologies can be clearly observed. In fact, the powder is mainly composed of compact spheres and cenospheres with a smooth surface, besides less regular particles, with a highly porous structure, which can be attributed to unburned coal [44]. The size of the spheres ranges between about 2 and 15 μm, whereas the irregular particles are typically larger, with an average size of about 25 μm. The higher magnification image (Figure 2D) evidences that the rounded grains are made by nanometric primary particles. The same ultrafine structure can be also detected in the struts of the porous irregular particles (Figure 2E).

The as-received RHA samples are black with some grey particles, resulting from different stages of the carbon combustion during burning of rice husk [30]. Their morphology is shown in Figure 2F–H, revealing particles with irregular shapes and a wide size distribution, in the range between about 20 and 400 μm. The higher magnification images allow one to observe the peculiar morphology of the fibrous flakes (Figure 2G) and the highly porous particles with a large internal surface area (Figure 2H), suitable in view of the dissolution of RHA under the alkaline medium.

The spherical morphology of FA powder explains the observed higher workability of the FA-R mixture compared to the HL-S and HL-R ones, as already reported in the literature [45,46]. Kong *et al.* [46] described the preparation of two series of geopolymers starting from metakaolin and fly ash, using solid-to-liquid (S/L) ratios of 0.8 and 3 for the former and latter powder, respectively. These authors justified this difference on the ground of the higher workability obtained with fly ash hollow spheres, compared to metakaolin powder.

**Figure 3:**
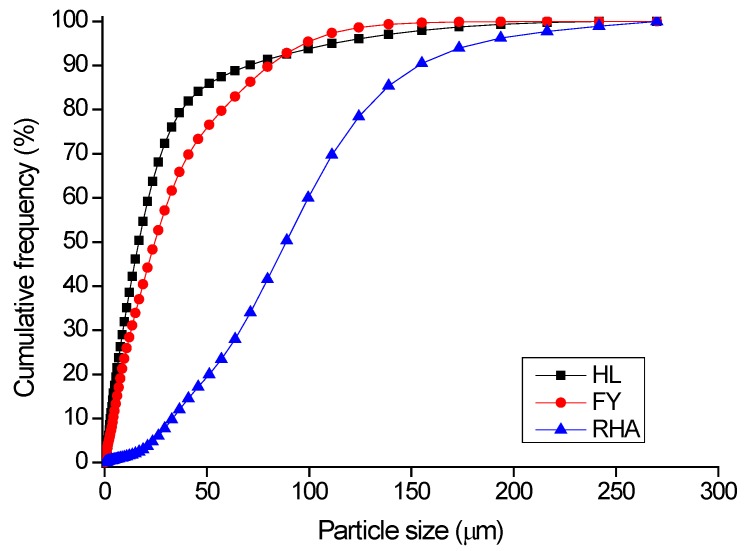
Particle size distribution (by volume) of raw materials: HL, FA and RHA.

In Figure 3, the particle size distribution (cumulative frequency, by volume) of the same powders is reported.

In Table 6, the particle sizes corresponding to 10% (d_10_), 50% (d_50_) and 90% (d_90_) of the cumulative distribution are collected. HL and FA are relatively fine powders, with a d_50_ of about 17 and 25 μm, respectively, and d_90_ values in the range 70–90 μm. The particle size distribution of RHA is displayed at larger values: the cumulative frequency distribution shows that 50% and 90% of the particles are smaller than about 90 and 155 μm, respectively.

### 3.2. Geopolymeric Pastes’ Characterization

#### 3.2.1. Dense Samples

Dense pastes of HL-S, HL-R and FA-R were obtained following the procedure described in the Materials and Methods section.

**Figure 4:**
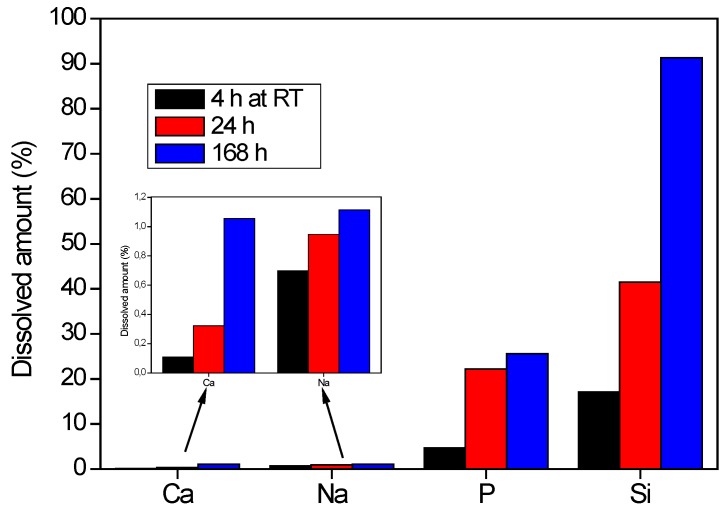
Effect of dissolution time Ca, Na, P and Si from RHA in alkaline medium.

In order to promote the solubilization of RHA in alkaline medium, the powder was sieved with a 125 mesh sieve, and the passing fraction was submitted to dissolution tests. Figure 4 collects the dissolution amount (at room temperature) of the main constituent elements of RHA (Si, P, Na and Ca) as a function of time. For all elements, a prolonged stirring time promotes their dissolution; the highest values were reached after 168 h of stirring in alkaline medium. The highest solubility was observed for silicon, reaching 91.3% of dissolution after one week. The solubility of phosphorous was moderate, reaching about 26% after the same stirring time. Na and Ca showed a very limited solubility (about 1%) in the described conditions, as better evidenced by the inset in Figure 4. This result was expected, as basic oxides (alkali and alkaline earth metal oxide) mainly dissolve in acid, whereas the dissolution of acid oxides (silica and phosphorous pentoxide) is promoted under alkaline conditions. However, these results clearly show that the dissolution of partially-crystallized RHA, and hence, the preparation of a proper potassium silicate solution, is a relatively long process, requiring at least one week.

The viscosity at 200 rpm was about 4500, 6600 and 6800 mPa∙s for HL-S, HL-R and FA-R, respectively. This condition was achieved by using a S/L of 2.5 for both HL-S and HL-R mixtures and a S/L of 4.3 for the FA-R composition, where the solid comprises the raw material (HL or FA), the silica powder (SiO_2_ or RHA) and the KOH pellets.

The density of the three kinds of samples is reported in Table 7. The substitution of nanosilica powder with RHA in the starting potassium silicate solution leads to a certain decrease (10%) of density. Instead, the higher density of FA-R can be related to the higher solid content of this mixture (S/L = 4.3) with respect to the HL-S and HL-R ones (S/L = 2.5) (Table 7).

**Figure 5:**
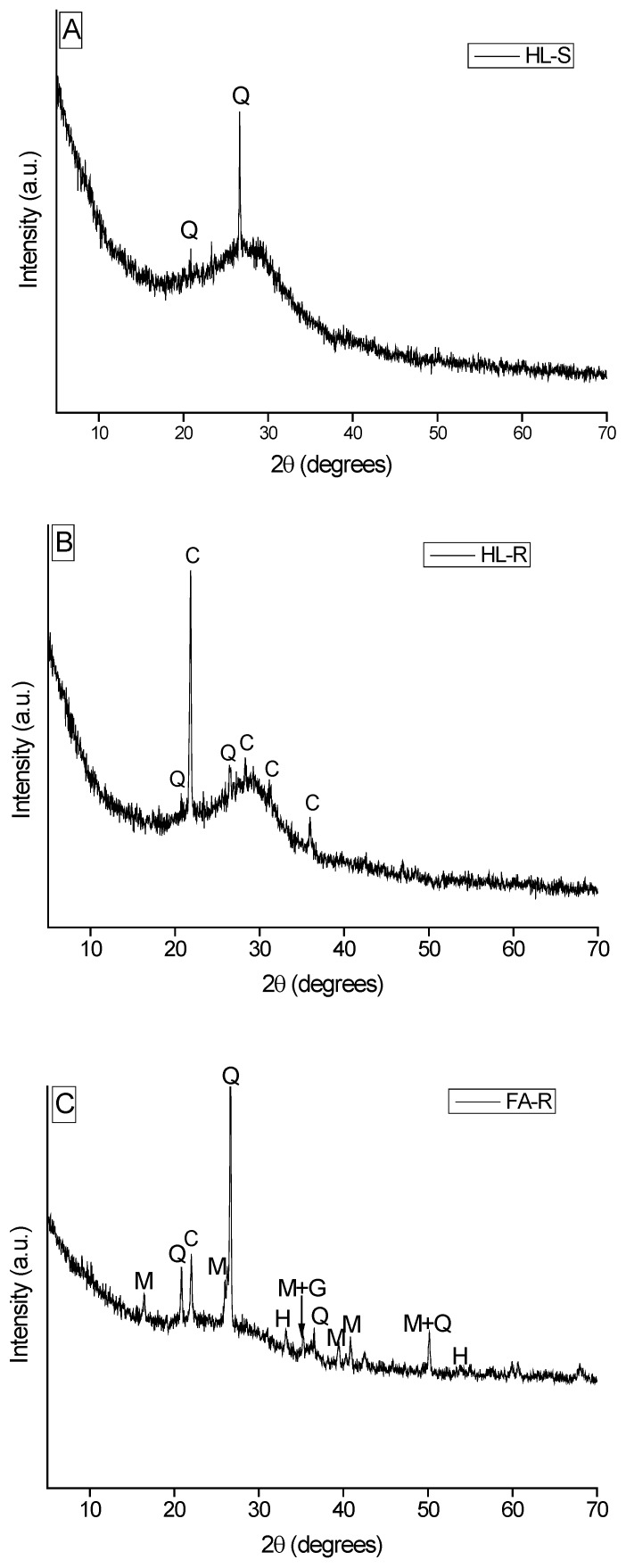
XRD patterns of geopolymers: (**A**) HL-S; (**B**) HL-R; (**C**) FA-R. (C = Cristobalite, Q = Quartz, G = magnetite, M = mullite).

In Figure 5, the XRD patterns of the geopolymer pastes are shown. All three materials are characterized by an important broad band, attesting to the prevalent amorphous structure of the geopolymers. Peaks related to crystalline phases can be, however, detected in all the samples, imputable to unreacted materials: in HL-S, the diffraction peaks of the quartz phase can be observed (A); in HL-R, beside quartz, the peaks of cristobalite are well evident (B); finally, in FA-R, the crystalline phases characterizing the fly ash powder (quartz, mullite, hematite and magnetite) are visible (C).

**Figure 6:**
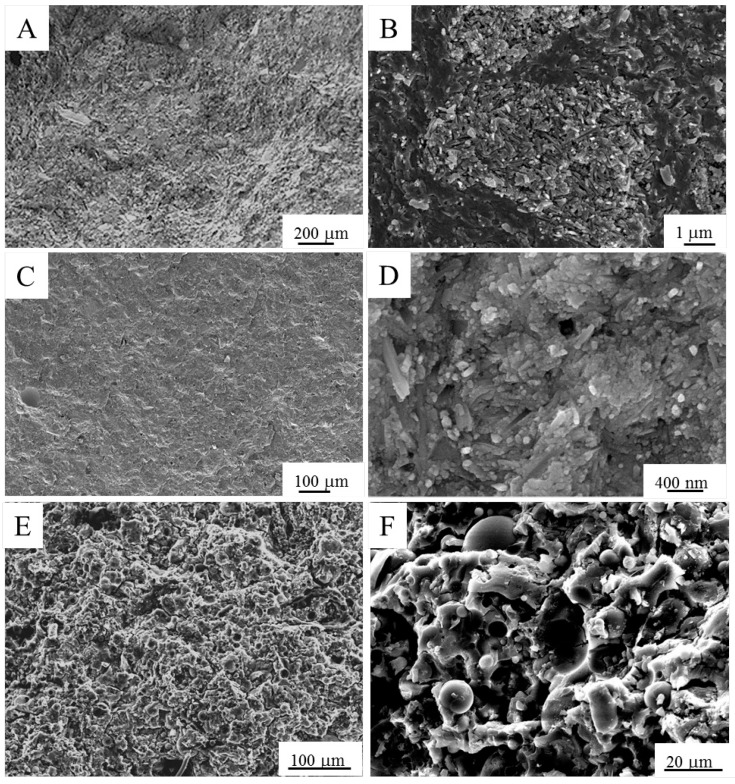
FESEM micrographs of the fracture surfaces of HL-S (**A**,**B**); HL-R (**C**,**D**) and FA-R (**E**,**F**).

In Figure 6, the microstructure of the samples can be seen. Both HL-based materials generally present a compact and glassy matrix (Figure 6A–C) in spite of the fact that in some areas, the primary halloysite particles (Figure 6B–D) are recognized, easily identified by their characteristic tubular morphology. Such areas suggest an incomplete reaction, in spite of a previous study by Zhang *et al.* [38] proving the high reactivity of calcined halloysite under an alkaline activator. Such reactivity, even higher than traditional metakaolin, was attributed to the randomly-displaced sequence of the individual two-sheet layers (Si–O tetrahedron and Al–O octahedron) along the *b* axis [47] of the tubular structure. Such a disordered structure makes the deconstruction of the Si–O–Si and Al–O–Si fast, since the alkaline solution can easily attack the tube from both the inside and outside surface. FA-R material showed a less compact microstructure as compared to HL-R and HL-S materials (Figure 6E). Here, although the matrix is continuous and relatively dense, voids and cracks are easily observed. In addition, unreacted or partially reacted fly ash spherical particles are clearly detectable (Figure 6F). It was already observed that a large proportion of fly ash does not completely react, especially the larger particles [48].

**Figure 7:**
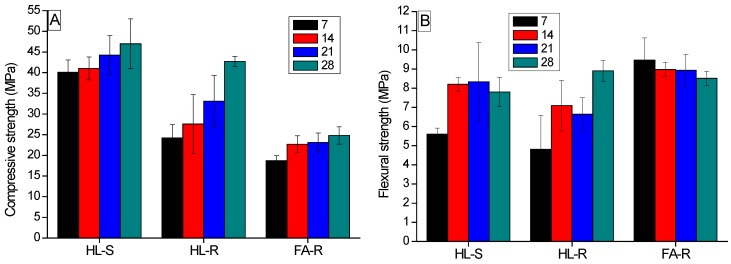
Compressive (**A**) and flexural (**B**) strength of dense MK-S, MK-R and FA-R specimens as a function of the curing time.

In Figure 7, the compressive (A) and flexural (B) strengths of the dense specimens are displayed. In general, a progressive increase of the strength by increasing the curing time was evidenced, as already reported in the literature [49]. However, by comparing the behavior of HL-S and HL-R, some differences can be observed. In fact, for HL-S, a relatively short curing time (7–14 days) is sufficient to reach high strength values, whereas HL-R requires a longer time (28 days) to achieve comparable (under compression) or even higher (under bending) values. Mechanical test results can be explained on the basis of the dissolution of the remaining silica from RHA: it is accomplished during the 28 days of curing, thus delaying the completion of the geopolymerization process. Most important, the current results show that RHA can be used as a valuable alternative to silica nanopowder to prepare the activator solution. In fact, the flexural and compressive strength values of HL-R reached about 9 and 43 MPa, respectively. The compressive strength here determined is in fair agreement with the few previous studies on geopolymers prepared by calcined clays activated with an RHA-based sodium or with potassium silicate solutions [50,51]. In fact, values of about 40 MPa were determined after curing the samples at room temperature for 28 days [51] or at 70 °C for 20 h, followed by a further 28 days of curing at room temperature [50].

FA-R shows a different dependence of the strength from the curing time. In fact, the maximum compressive and flexural strength values were achieved during the first 7–14 days, and the subsequent curing does not significantly increase the mechanical performance. Here, differently from previous samples, the curing was carried out at 50 °C, which could promote the dissolution of residual silica, the potassium alumino-silicate formation and, hence, the geopolymerization process.

HL-R samples achieved a high flexural strength: after 28 days of curing, the values are comparable, or even higher, than those of HL-S mixtures. On the opposite side, the compressive strength of FA-R is significantly lower than that of HL-S and HL-R samples. It should be considered that the same alkaline solution was used for the three formulations, but in different ratios, which was determined on the ground of the pastes’ workability. Accordingly, the SiO_2_/Al_2_O_3_ molar ratio, which is known to rule the mechanical performance of geopolymers [12], is significantly different in the three mixtures. HL-S and HL-R, prepared with a S/L ratio of 2.5, are characterized by a Si/Al molar ratio of 1.7. It was demonstrated that, when metakaolin is used as a raw material, the Si/Al ratio should be increased over 1.65 to achieve high mechanical strength, in fair agreement with this study [52]. On the contrary, the best workability of FA-R was achieved at a S/L ratio of 4.3, which corresponds to a water/fly ash mass ratio of 0.31, close to the values used by Li *et al.* [45] to develop high strength pastes and mortars. However, in this condition, the Si/Al molar ratio of the mixture was 2.7, which is higher than the optimal values for fly ash geopolymers to achieve high mechanical strengths [53]. Chindaprasirt *et al.* [48] showed that a high Si/Al ratio corresponds to low mechanical strengths. Duxson *et al.* [20] demonstrated that the decrease in strength, obtained when the silica content is higher than the optimum values, could be linked to the amount of unreacted material in the specimens, which act as defect sites.

In general, a direct comparison between the results of this study and literature data is difficult, since very few works have previously discussed the mechanical properties of fly ash geopolymers activated by a RHA-based solution. Detphan and Chindaprasirt [54] investigated the strength of geopolymer mortars with RHA/FA mass ratios of 0/100, 20/80, 40/60 and 60/40, activated with sodium hydroxide and sodium silicate and cured at 60 °C, showing compressive strengths between 12.5 and 56.0 MPa, dependent on the ratio of FA/RHA, the RHA fineness and the ratio of sodium silicate to NaOH. Chaiyapoom *et al.* [55] developed geopolymers based on fly ash activated by sodium hydroxides. RHA was used as a raw material to partially replace the fly ash, inducing a progressive increase of the bending strength up to a maximum of about 8 MPa.

#### 3.2.2. Porous Samples

**Figure 8:**
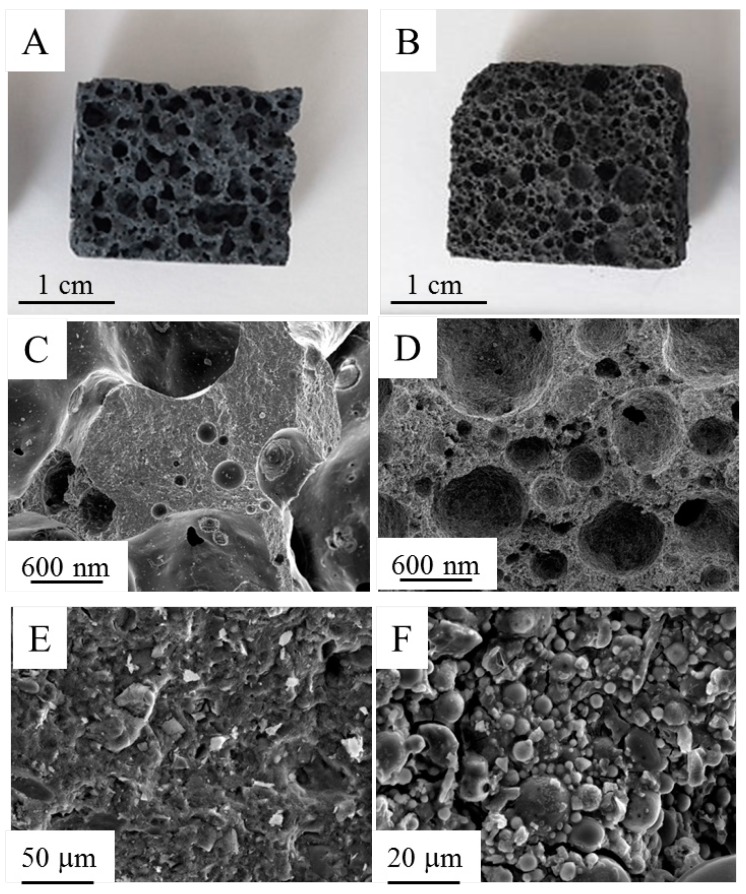
Digital photographs and related microstructure of: (**A**,**C**,**E**) HL-R; (**B**,**D**,**F**) FA-R. Both porous samples contain Al at 0.1%.

Starting from the more innovative compositions, HL-R and FA-R, macroporous samples were produced at different foaming agent amounts (Al in the range 0.05%–0.3%). In Figure 8, the digital photographs of the fracture surfaces (Figure 8A,B) and related microstructures of foamed samples at 0.1% of Al are depicted. For both samples, the detail of the porous structure (Figure 8C,D) and of the struts between the pores (Figure 8E,F) is shown.

**Figure 9:**
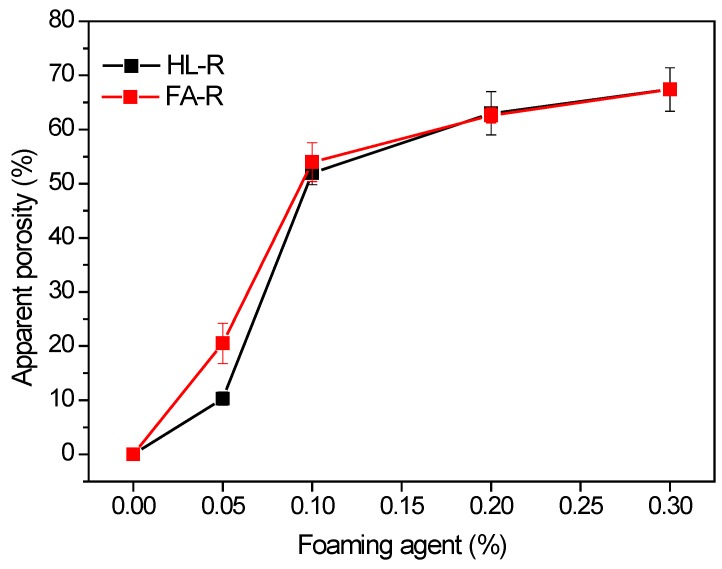
Apparent porosity of HL-R and FA-R samples as a function of the foaming agent amount.

Figure 9 shows the apparent porosity as a function of Al amount. For both materials, the addition of low amounts (0.05% and especially 0.1%) of foaming agent is very effective in increasing the apparent porosity, leading to values of 52%–54% at 0.1% of Al for HL-R and FA-R materials, respectively. On the opposite side, further additions induce a limited increase of porosity: with 0.2% of Al, both samples reach an apparent porosity of 63%, whereas with 0.3% Al, the apparent porosity increases up to 67%. It should be observed that, with the sole exception of the samples containing 0.05% of Al, in all other cases, the foaming agent has the same effect of decreasing the density in the two compositions.

**Figure 10:**
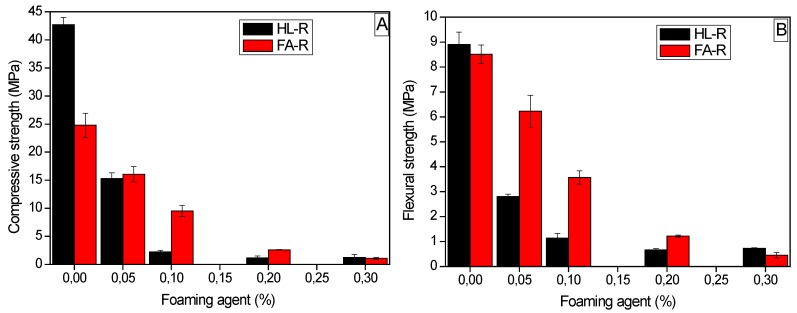
Evolution of the (**A**) compressive and (**B**) flexural strength of HL-R and FA-R as a function of the foaming agent.

The evolution of the compressive (Figure 10A) and of flexural (Figure 10B) strength as a function of the foaming agent content shows that for HL-R, the decrease of both compressive and flexural strength is particularly pronounced for 0.05% and 0.1% of Al, corresponding to the neat increase of the apparent porosity. Instead, in the case of FA-R, a more gradual decrease of both mechanical properties by increasing the foaming agent amount is observed. As already commented, the compressive strength of FA-R is significantly lower than the HL-R one. However, when porous samples are concerned, FA-R shows comparable or even higher strength than HL-R. For 0.1% of Al, FA-R strength is more than four times higher than the HL-R one; however, this difference is annihilated at higher porosity amounts, becoming comparable at 0.3% of Al. A similar behavior was observed for the flexural strength, where FA-R shows significantly increased strength, particularly at 0.05% and 0.1% of Al, whereas at higher foaming agent amounts, this difference decreases.

### 3.3. Preliminary Evaluation of Global Warming Impact

A very preliminary study of the Global Warming Impact (GWP) of the three different compositions was carried out on the ground of data available in the literature. The aim was to verify and to quantify the reduction of CO_2_ emissions when natural/synthetic raw materials used for the production of geopolymers are substituted with waste products (namely: substitution of halloysite with fly ash and of amorphous nanosilica powders with rice husk ash). In this preliminary work, the contribution of transport and curing on the total GWP were not considered, therefore following a “cradle-to-gate” approach.

In Table 8, the minimum and maximum literature values found for each component are reported.

Even if calcined clays are frequently used to produce geopolymers, there is a high variability of data in the peer-reviewed literature concerning the environmental impact of this material. In the study of Heath *et al.* [56], GWP values ranging from 0.09–0.43 kg CO_2_-eq/kg were reported. Both of these values considered the clay extraction and the extra amount of unfired clay that needs to be burnt to reach 1 kg of metakaolin. The lower value came from the fact that biogas from agricultural waste was used to calcine the clay instead of heavy fuel [57]. In fact, as analyzed in the recent study of Habert [57], the environmental impact of metakaolin is highly sensitive to the fuel source used for calcination than the production process itself.

As discussed before, in order to optimize the molar ratio of the geopolymer composition, silica nanopowder was added, as commonly done in the literature [26]. In terms of environmental impact, the use of nanosized powders instead of a common amorphous silica could have heavy consequences. Nevertheless, a gap exists in life cycle analysis (LCA) studies in the area of nanotechnology. The environmental assessment of the silica nanopowder was reported only in the study of Roes *et al.* [58] in which the environmental impact of the four different types of nanosilica manufactured at industrial scale (colloidal silica, silica gel, precipitated silica and pyrogenic silica) was estimated. The differences in the GWP values between these products are all associated with the manufacturing process. Since silica gel and precipitated silica are sold as dried powders (as the material used in the production of geopolymers reported in the present study), their minimum and maximum values were considered as a reference.Data related to the GWP of potassium hydroxide are fewer than the information provided for sodium hydroxide. In any case, as specified in Heath [56], KOH is manufactured using a similar process as NaOH.

For this reason, the maximum and minimum values here used are referred to as sodium hydroxide production and were both deduced from the study of Habert [57]. The minimum value is related to the production of NaOH in Europe, while the higher value refers to an extra-European production. The variability of these data is rather imputable to the choice of the electricity mix rather than to the production process.

The question of the value of the environmental load of by-products, such as fly ash, has been discussed by several authors, as summarized in some previous papers [25,57,59]. As again argued in [57], the current status would be to consider these materials as waste and to allocate no impact from the main process, but only to the process directly linked to their transformation from a waste to valuable precursor. Since in this study the transportation impacts were excluded and fly ash and RHA powder were used as received, their GWP was here considered as null.

**Figure 11:**
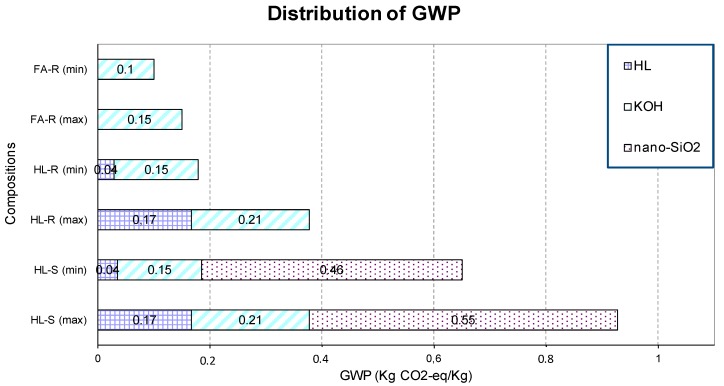
Distribution of GWP for each component of the three compositions, considering the minimum and maximum values found in the literature.

The graph reported in Figure 11 shows the allocation of GWP by geopolymer ingredients for each formulation.

The composition HL-S results in the highest GWP due to the contribution of all the components. The value ranges between 0.65 and 0.9 kg CO_2_-eq/kg, depending on the data source. In particular, the important contribution of nanosilica can be easily observed, contributing more than the sum of KOH and calcined clay emissions. When the nanosilica is substituted with RHA, the GWP values strongly decrease: the GWP of HL-R ranges between a total value of 0.18 and 0.46 kg CO_2_-eq/kg.

The GWP values reached with the compositions HL-R (min) and FA-R (max) are comparable. In fact, in the first case, the impact of the clay calcined with agricultural waste is quite negligible and comparable to the value of FA. The difference is due to the amount of alkaline activator used and the value considered for it.

In any case, the lowest environmental impact was obtained in the last mixture, in which FA substituted halloysite as the raw material, RHA substituted the nanosilica powder in the solution and the amount of potassium silicate solution used was lower with respect to the previous two compositions, due to the highest workability of FA-R. Ultimately, only KOH gives a considerable contribution to the GWP, while the other components do not provide any impact. This composition reached a total value of 0.1–0.15 kg CO_2_-eq/kg. In any case, it seems quite difficult to hold a clear opinion on the environmental impact of the mixtures here discussed in comparison with other geopolymers presented in the literature, due to the high variability of the formulations. Similarly, the comparison of the achieved results with Portland cement is not so immediate, because nowadays, more and more concrete is produced with blended cement. However, generally speaking, the reported emission factor for cement production is 0.82 kg CO_2_-eq/kg for OPC [25].

As highlighted in the incipit of the paragraph, transport contribution was not considered in this preliminary evaluation. This aspect, as reported in the study of McLellan *et al.* [24], could be considered the main weak point for geopolymers, in particular when compared to Portland cement. In fact, for concrete, the distances are generally short because the production plants are rather spread out in the territory. Nevertheless, in view of a further improvement of this work, it should be interesting to specify that the RHA used in this research is locally produced. Moreover, in our region, a kaolin quarry that could supply the raw material also exists, limiting the use of imported raw materials, which obviously have a heavy impact in terms of CO_2_ emissions.

## 4. Conclusions

This paper demonstrated the feasibility of two industrial wastes, fly ash (FA) and rice husk ash (RHA), as raw materials for the production of innovative geopolymeric pastes. In particular, this study explores the use of RHA as a valuable alternative to silica nanopowder to prepare the activator solution.

Three typologies of samples were produced: (i) a calcined clay (Halloysite, HL) activated with potassium hydroxide and nanosilica, used as the reference sample (HL-S); (ii) halloysite activated with RHA dissolved into KOH solution (HL-R); (iii) FA activated with the alkaline solution realized with RHA (FA-R).

Substitution of the silica nanopowder with RHA in the starting potassium silicate solution led to a certain decrease (10%) of density. Instead, the higher density of FA-R could be related to the higher solid content of this mixture (S/L = 4.3) with respect to the HL-S and HL-R ones (S/L = 2.5). In fact, the rounded morphology of the fly ash powders showed a positive role in the mixture workability, as compared to the halloysite-based mixtures. Thus, the highest workability of FA-R allowed us to increase its solid loading, moving from the S/L value of 2.5 in HA-S and HA-R, to 4.3 in FA-R.

For HL-S, a relatively short curing time (7–14 days) was sufficient to reach high strength values, whereas HL-R required a longer time (28 days) to achieve comparable (under compression) or even higher (under bending) values. In fact, HL-R reached a flexural strength of about 9 MPa and a compressive strength of 43 MPa. On the contrary, the compressive strength of FA-R was significantly lower than the HL-R one, probably due to some cracks observed in the microstructure and to the Si/Al molar ratio, which was higher than the literature optimal values. However, when porous samples were concerned, FA-R showed comparable or even higher strength than HL-R. In this case, the macro-porosity of the samples governs the material’s failure, as observed with other engineering ceramic foams [60]. Anyway, RHA addition to the geopolymeric composition proved to be effective either to produce dense or porous samples. In a future work, the thermal properties of the new, waste-derived porous materials will be assessed.

Finally, to quantify the benefit on the environmental impact associated with the use of waste materials in the production of geopolymers, a preliminary evaluation of the embodied carbon was calculated for the three investigated formulations. The composition HL-S presented the highest GWP due to the contribution of all the components, with values ranging between 0.65 and 0.9 kg CO_2_-eq/kg, depending on the data source. In particular, the important contribution of nanosilica was easily evidenced, contributing more than the sum of KOH and calcined clay emissions. When the nanosilica was substituted with RHA, the GWP values strongly decreased and ranged between 0.18 and 0.46 kg CO_2_-eq/kg. However, the lowest environmental impact was obtained when FA substituted halloysite as the raw material, RHA substituted the nanosilica powder in the solution and the amount of potassium silicate solution used was lower with respect to the previous two compositions, due to the highest workability of FA-R. At this point, only KOH gives a considerable contribution to the GWP, while the other components did not provide any impact. This composition reached a total value of 0.1–0.15 kg CO_2_-eq/kg emissions.

## Figures and Tables

**Table 1:** Composition and designation of the dense samples.

Designation	Raw Material	Alkaline Solution
HL-S	Calcined halloysite	KOH + SiO_2_
HL-R	Calcined halloysite	KOH + RHA
FA-R	Fly ash	KOH + RHA

**Table 2:** Designation and chemical composition (expressed as wt %) of the geopolymer pastes (HL-S, HL-R, FA-R) and of the macroporous samples.

Sample	Type of Sample	HL	FA	H_2_O	KOH	SiO_2_	RHA	Al
HL-S	Dense	41.84	-	28.08	15.89	14.18	-	-
HL-R	Dense	41.84	-	28.08	15.89	-	14.18	-
FA-R	Dense	-	60.98	18.84	10.66	-	9.52	
HL-R with 0.05%Al	Porous	41.83	-	28.07	15.88	-	14.17	0.05
HL-R with 0.1%Al	Porous	41.81	-	28.05	15.87	-	14.17	0.1
HL-R with 0.2%Al	Porous	41.77	-	28.02	15.86	-	14.15	0.2
HL-R with 0.3%Al	Porous	41.72	-	28.00	15.84	-	14.14	0.3
FA-R with 0.05%Al	Porous	-	60.95	18.82	10.65	-	9.51	0.05
FA-R with 0.1%Al	Porous	-	60.91	18.82	10.65	-	9.51	0.1
FA-R with 0.2%Al	Porous	-	60.86	18.80	10.64	-	9.50	0.2
FA-R with 0.3%Al	Porous	-	60.80	18.78	10.63	-	9.49	0.3

**Table 3:** Chemical composition of calcined HL.

Oxide	wt %
SiO_2_	54.20
Al_2_O_3_	44.10
Fe_2_O_3_	0.81
P_2_O_5_	0.48
CaO	0.17
SrO	0.15
NiO	0.07
Total	99.98

**Table 4:** Chemical composition of FA.

Oxide	wt %
SiO_2_	59.80
Al_2_O_3_	25.00
Fe_2_O_3_	9.43
K_2_O	2.54
CaO	2.22
SO_3_	0.78
SrO	0.15
ZrO_2_	0.04
Total	99.96

**Table 5:** Chemical composition of RHA.

Oxide	wt %
SiO_2_	91.50
K_2_O	4.14
P_2_O_5_	1.48
CaO	1.19
SO_3_	1.03
NaO	0.65
Total	99.99

**Table 6:** Particle sizes at 10% (d_10_), 50% (d_50_) and 90% (d_90_) of the cumulative distribution for HL, FA and RHA powders.

Powder	d_10_ (μm)	d_50_ (μm)	d_90_ (μm)
HL	1.6	17.2	68.9
FA	4.1	24.8	81.4
RHA	32.6	89.0	155.2

**Table 7:** Density of the dense pastes HL-S, HL-R and FA-R pastes.

Sample	Density (g/cm^3^)
HL-S	1.50 ± 0.02
HL-R	1.35 ± 0.03
FA-R	1.76 ± 0.03

**Table 8:** Minimum and maximum values of CO_2_ eq for each component used in the geopolymeric formulations and the indication of the data sources. GWP, global warming potential.

Component	GWP100 min (kg CO_2_-eq/kg)	Ref.	GWP100 max (kg CO_2-eq_/kg)	Ref.
Calcined clay (metakaolin)	0.09	[47,48]	0.42	[47,48]
Fly ash	0	-	0	-
Nanosilica	3.48	[49]	4.12	[49]
Potassium hydroxide	0.99	[48]	1.43	[48]
RHA	0	-	0	-

## Abbreviations

The following abbreviations are used in this manuscript:

FA	Fly ash
RHA	Rice husk ash
XRD	X-ray diffraction
PDF	Powder data file
XRF	X-ray fluorescence
ICP-AES	Inductively-coupled plasma-atomic absorption spectrometer
PTFE	Polytetrafluoroethylene
FE-SEM	Field emission-scanning electron microscope
EC	Embodied carbon
S/L	Solid/liquid
